# Automatic robotic doppler sonography of leg arteries

**DOI:** 10.1007/s11548-024-03235-7

**Published:** 2024-07-25

**Authors:** Jonas Osburg, Alexandra Scheibert, Marco Horn, Ravn Pater, Floris Ernst

**Affiliations:** 1 Institute for Robotics and Cognitive Systems, University of Luebeck, Ratzeburger Allee 160, Luebeck, 23562 Germany; 2 Clinic for Surgery, Division for Vascular and Endovascular Surgery, University Clinic Schleswig-Holstein Campus Luebeck, Ratzeburger Allee 160, 23538 Luebeck, Germany

**Keywords:** Robotic ultrasound, 3D ultrasound, Duplex sonography, Doppler sonography, Peripheral arterial disease, Visual servoing

## Abstract

****Purpose**:**

Robot-assisted systems offer an opportunity to support the diagnostic and therapeutic treatment of vascular diseases to reduce radiation exposure and support the limited medical staff in vascular medicine. In the diagnosis and follow-up care of vascular pathologies, Doppler ultrasound has become the preferred diagnostic tool. The study presents a robotic system for automatic Doppler ultrasound examinations of patients’ leg vessels.

****Methods**:**

The robotic system consists of a redundant 7 DoF serial manipulator, to which a 3D ultrasound probe is attached. A compliant control was employed, whereby the transducer was guided along the vessel with a defined contact force. Visual servoing was used to correct the position of the probe during the scan so that the vessel can always be properly visualized. To track the vessel’s position, methods based on template matching and Doppler sonography were used.

****Results**:**

Our system was able to successfully scan the femoral artery of seven volunteers automatically for a distance of 20 cm. In particular, our approach using Doppler ultrasound data showed high robustness and an accuracy of 10.7 (±3.1) px in determining the vessel’s position and thus outperformed our template matching approach, whereby an accuracy of 13.9 (±6.4) px was achieved.

****Conclusions**:**

The developed system enables automated robotic ultrasound examinations of vessels and thus represents an opportunity to reduce radiation exposure and staff workload. The integration of Doppler ultrasound improves the accuracy and robustness of vessel tracking, and could thus contribute to the realization of routine robotic vascular examinations and potential endovascular interventions.

## Introduction

Ultrasound (US) imaging offers several advantages for diagnostic and interventional procedures. These include freedom from ionizing radiation, good soft tissue contrast, high flexibility, and real-time image acquisition. Furthermore, ultrasound can provide real-time volumetric image data (4D US) [1] and has proven to be a valid imaging modality in the field of interventional procedures [2]. Additionally, Doppler ultrasound, also referred to as duplex sonography, provides the examiner with additional information on the kinetics of blood flow, which is crucial, for example, in the assessment of hemodynamically relevant vascular stenosis. Duplex sonography has become the preferred diagnostic tool, especially in the diagnosis and follow-up care of vascular pathologies [3]. As the incidence of vascular diseases has steadily increased over the years [4], the number of patients requiring pre- and post-examination for peripheral arterial diseases continues to grow. Thus, there will be a significant increase in demand for routine standardized duplex sonography of the leg vessels. A major challenge in obtaining high-quality US images is the strong dependence on the clinician’s experience. This leads to a reduced ability to reproduce the recorded US images consistently [5, 6]. Furthermore, there are ergonomic burdens on the sonographer [7, 8] and a general shortage of staff in clinics. Automated systems for US scanning can be an opportunity to reduce the problems mentioned above. By combining US imaging and a robotic system, precise and repeatable images can be acquired in an automated manner [9], while ergonomically relieving sonographers and counteracting staff shortages. Thus, a robotic system to perform automatic ultrasound scans could provide reliable support in the care of patients with vascular diseases.

## Related work

Various robotic US systems for automated scanning of vascular structure are described in the literature [9–11]. In [12] a robotic arm was used to scan the lower limb artery. The vessel lumen was segmented from the B-mode image using a fast-marching method and the position of the probe was adjusted accordingly to keep the artery continuously in the center of the image. Haxthausen et al. also presented an approach in which a robot successfully followed the peripheral artery of a phantom automatically. The vessel center was determined either using a neural network [13] or a template-matching method [14]. Jiang et al. [15] developed a fully robotic system for detecting tubular structures based on a neural network for segmentation. In addition, the US probe was automatically aligned with the normal direction of the target structure, while the vessel structure was held in the center of the US view. However, the robot systems described above were only evaluated based on phantom experiments.

A volunteer study on the realization of automatic carotid scan procedures using imitation learning was presented in [16]. In addition, a volunteer study was done in [17] to demonstrate an approach to improving the accuracy and robustness of vessel segmentation using Doppler US images. To the best of our knowledge, this is only one study in the literature using Doppler ultrasound for robot-guided examinations, although Doppler ultrasound has become the preferred diagnostic tool, especially in the diagnosis and follow-up care of vascular pathologies [3]. The most recent system for fully automated, robot-assisted 3D ultrasound image acquisition for arteries is presented in [18] and was also validated by a volunteer study. A neural network with a u-net structure was used to determine the vessel center, while the in-plane rotation of the probe was corrected by a US confidence map.

In this paper, we present another robotic system for automatic scanning of the leg artery. We extend the state of the art by using a 3D ultrasound probe and Doppler US to track the vessel’s position. We use the X-Plane mode to record the Doppler data, as we achieve a reasonably acceptable recording frequency of 10 Hz and the information content of the recorded data is considerably higher than in the pure 2D case due to the additional preservation of the longitudinal section of the vessel. An adapted impedance control is used to ensure patient safety and proper contact with the US probe. We validate our system by performing a study with seven volunteers.

## Methods

The robotic system described in this paper aims to enable automatic US scans of the femoral artery. The US probe must be moved with a certain contact force while the position is adjusted to keep the vessel fully visible. This requires system calibration, the use of compliant robot control, and tracking of the artery position in the US images.

### System calibration

**Fig. 1:**
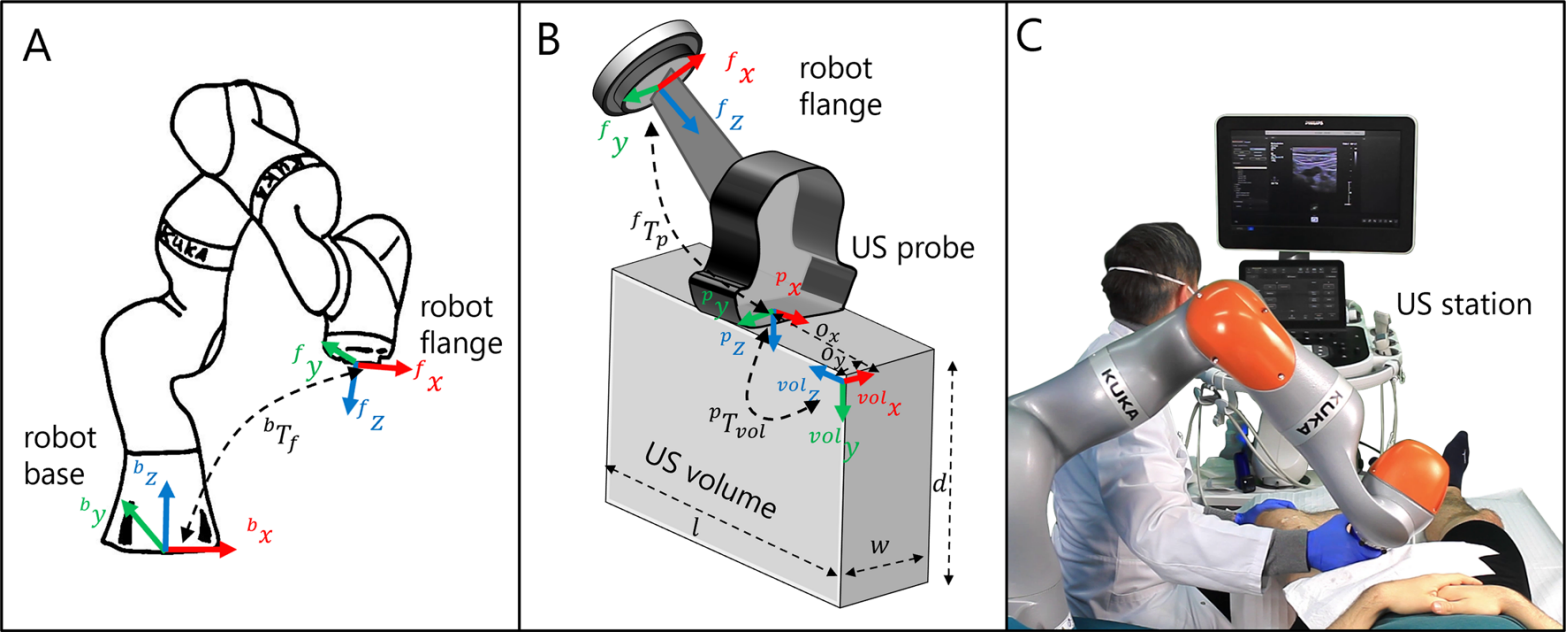
**A** Visualization of the base and flange frame of the robot as well as the transformation $^bT_f$. **B** Geometric representation of transformations $^fT_p$ and $^pT_{vol}$. **C** Experimental setup of the volunteer study

To control the position of the robot based on a US volume or a sectional plane of this volume, the transformation matrix $^bT_{\text {vol}}$ must be determined. This matrix transforms pixel positions from an US volume {vol}, into the base frame {*b*} of the robot. The frames involved in this process are shown in Fig. 1.

Consequently, the calculation of $^bT_{\text {vol}}$ can be performed as follows:

1 $$\begin{aligned} ^bT_{\text {vol}} = \ ^bT_f \ ^fT_p \ ^pT_{\text {vol}} \end{aligned}$$

where $^jT_i \in \mathbb {R}^{4\times 4}$ is the homogeneous transformation matrix to transfer the position from frame {*i*} to frame {*j*}. The probe tip’s frame is centered on the US probe and referred to as {*p*}. The frame of the robot flange is denoted as {*f*}. Transformation $^bT_f$ can be derived from the measured joint angles and the forward kinematics of the robot. Transformation $^fT_p$ can be achieved from the CAD model of the probe holder and the probe attached to it. To determine the transformation $^pT_{\text {vol}}$, a spatial calibration can be performed. This is done either by using customized phantoms [19–21] or by employing image-based methods [22]. The resulting transformation $^pT_{\text {vol}}$ contains the homogeneous rigid transformation $^pT_{\text {vol,grd}}$ from the probe tip’s frame to the image frame as well as the image spacing represented as affine transformation $S \in \mathbb {R}^{4\times 4}$ with spacing factors $s_x, s_y$ and $s_z$ for the respective axes:

2 $$\begin{aligned} ^pT_{\text {vol}} = \ \underbrace{\begin{pmatrix} R &{} \begin{matrix} o_x \\ o_y \\ o_z \end{matrix} \\ \begin{matrix} 0 &{} 0 &{} 0 \end{matrix}&1 \end{pmatrix}}_{\begin{array}{c} ^pT_{\text {vol,grd}} \end{array}} \ \underbrace{\begin{pmatrix} s_x &{} 0 &{} 0 &{} 0 \\ 0 &{} s_y &{} 0 &{} 0 \\ 0 &{} 0 &{} s_z &{} 0 \\ 0 &{} 0 &{} 0 &{} 1 \end{pmatrix}}_{S} \end{aligned}$$

The transformation $^pT_{\text {vol,grd}}$ consists of a rotation matrix *R* describing the geometric relationship between the orientation of the probe tip’s frame {*p*} and the orientation of the US volume frame {vol} as well as a translational offset $o = (o_x,o_y,o_z)^T$ between the origins of the frames (see Fig. 1B). However, precise spatial calibration is an error-prone, time-consuming step. In addition, precise spatial calibration is not always required. For example, methods for 3D volume reconstruction are also possible without perfect spatial calibration, either by directly merging 3D US volume data by volume-to-volume registration methods [23], or by using inertial measurement units to track the probe’s orientation [24]. Furthermore, diagnostic applications such as the one shown in this study can be performed without precise spatial calibration. For this reason, we use a simplified option by determining an approximate transformation matrix $^pT_{\text {vol,smp}}$, which considerably simplifies the workflow and improves usability. The aim of robotic US scans based on visual servoing is to move the probe depending on the US volume or image. Instead of a movement to a specific position, it is often sufficient to move the probe in a certain direction, e.g. in the direction of the vessel to be tracked. Thus, the essential parameter is the rotation matrix *R* between the orientation of the probe tip’s frame {*p*} and the US volume frame {vol}. This can be determined experimentally by positioning the probe attached to the robot flange in such a way that the cross-section of the artery (or any other easy trackable structure) is visible in the $^\mathrm{{vol}}xy$-plane of the US volume. Its initial position is determined by manually selecting the cross-section from the US image. The probe is then moved transversely for a small distance in the positive $^py$-direction of the probe tip’s frame. The displacement of the vessel in the $^\mathrm{{vol}}xy$-plane can either be recognized manually by the operator or can be tracked by a template matching algorithm described in Section “Vessel template matching”. Finally, the difference between the $^\mathrm{{vol}}x$-values of the start and end position of the vessel is calculated. If the difference is positive, the $^\mathrm{{vol}}x$-axis and the $^py$-axis point in the opposite direction, resulting in the rotation matrix $R_1=(0,0,-1;-1,0,0;0,1,0)^T$. Correspondingly, if the difference is negative, the $^\mathrm{{vol}}x$-axis and the $^py$-axis point in the same direction, resulting in the rotation matrix $R_2=(0,0,1;1,0,0;0,1,0)^T$. The offset values $(o_x,o_y,o_z)^T$ of the calibration matrix $^pT_\mathrm{{vol,smp}}$ can be derived geometrically as the interface of the US station provides us with the size of the US volume (length *l*, width *w*, depth *d*, see Fig. 1B) in pixels as well as the corresponding image spacing $s_x,s_y$ and $s_z$. This allows us to calculate the offsets $o_x$ and $o_y$ between the probe and volume frames, assuming that the volume is located in the center under the probe.

3 $$\begin{aligned}{} & {} o_x = \frac{l}{2} \ s_x \end{aligned}$$

4 $$\begin{aligned}{} & {} o_y = \frac{w}{2} \ s_y\end{aligned}$$

5 $$\begin{aligned}{} & {} o_z = 0 \end{aligned}$$

The offset $o_z$ is neglected since it is only a minimum distance in $^pz$-direction between the origin of the probe frame and the US volume frame, as defined by the arrangement of the transducer elements in the probe. This results in the following transformation matrix for the case $R=R_1$ as shown in Fig. 1B:

6 $$\begin{aligned} ^pT_\mathrm{{vol,smp}} = \begin{pmatrix} 0&{}0&{}-s_z&{}\frac{l}{2} s_x \\ -s_x&{}0&{}0&{}\frac{w}{2} s_y\\ 0&{}s_y&{}0&{}0\\ 0&{}0&{}0&{}1 \end{pmatrix} \end{aligned}$$

The described approximate calibration method offers the advantage of requiring no additional time for spatial calibration with a phantom, as long as the probe remains attached to the end effector in the same position. All necessary values for Eq. (6) are obtained from the ultrasound station’s interface, allowing for complete automation of the process.

### Robot control

To apply a certain contact force between the probe and the patient, a compliant control based on the internal joint torque sensors of the robot was implemented. The controller aims to apply a force $f_d$ in the direction of the probe’s $z-$axis (1 DoF). At the same time, a translational movement in the probe frame’s $x-$ and $y-$axes as well as a rotational movement around the $x-,y-$ and $z-$axes (5 DoF) can be enabled. The required joint velocities to enable this behaviour are calculated as

7 $$\begin{aligned} \dot{q}_c=(J(q)^T F_d -\tau _\textrm{ext}) D^{-1} + J^{\dagger }V_d \end{aligned}$$

where $J(q)^T \in \mathbb {R}^{7\times 6}$ is the transposed Jacobian matrix for the measured joint positions *q* and $\tau _\textrm{ext}$ represent the measured external joint torques. The desired wrench is defined as , where $f_d$ is defined as the applied force. $D \in \mathbb {R}^{7\times 7}$ is a diagonal damping matrix, with damping factors $d_i$ representing the diagonal entries. $J^{\dagger }$ represents the Moore-Penrose-Inverse of the Jacobian, denoted as $J^{\dagger } = J^T(JJ^T)^{-1}$. The desired twist is defined as $V_d = (v_d, w_d)^T = (\dot{x}, \dot{y}, 0, \dot{\phi }, \dot{\theta }, \dot{\psi })^T$, where $v_d = (\dot{x}, \dot{y}, 0)^T$ represents the translational motion and $w_d =(\dot{\phi }, \dot{\theta }, \dot{\psi })^T$ the rotational motion around the $x-,y$ and $z-$axes. The joint positions resulting from Eq. (7) are used in the target values for the robot’s joint impedance controller to increase stability during contact with the environment.

### Image analysis

A two-step approach is used for artery tracking. In the first step, possible vessel positions are determined using image processing. This is done either by searching for the vessel position using template matching (see Section “Vessel template matching”) or by extracting the red-colored blood flow in the US Doppler images (see Section “Vessel doppler signal”). In the second step, the vessel positions identified are then checked for plausibility with the previous time step (see Section “Vessel tracking”).

#### Vessel template matching

**Fig. 2:**
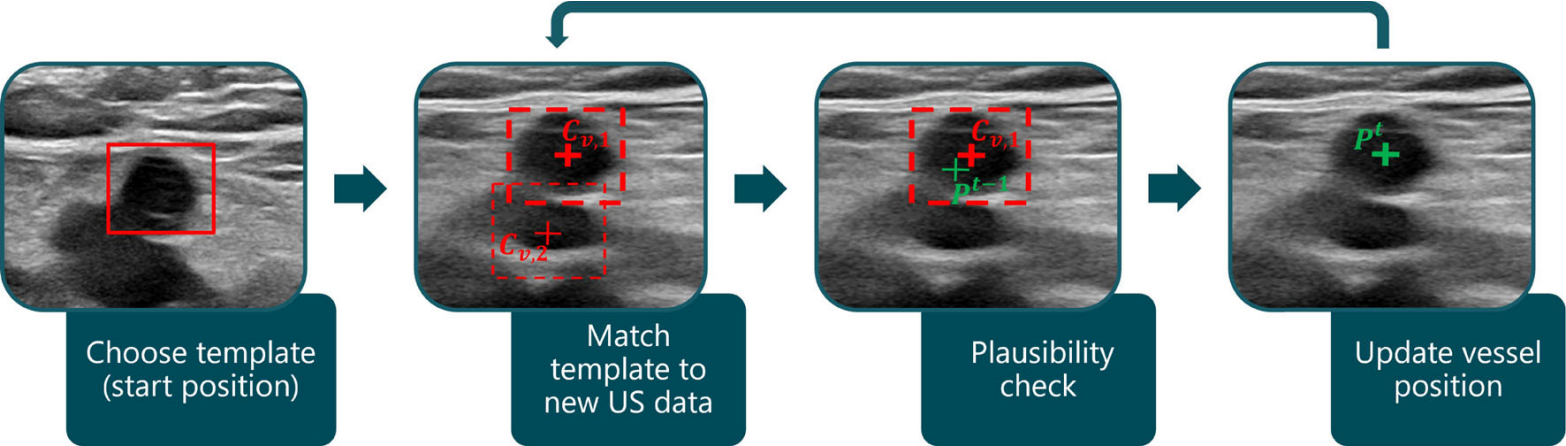
Procedure of template matching vessel tracking. The vessel template is selected at the scan’s start position. This template is then searched for in the new US images during the scan. The thicker dashed red box indicates the potential position $C_{v,1}$ with the highest correlation coefficient. This position is then compared with the vessel’s position $P^{t-1}$ from the previous time step

The procedure of our first approach, which uses a template matching algorithm to find the vessel’s position, is illustrated in Fig. 2.

This involves the physician or operator of the system selecting the cross-section of the artery at the start position of the scan. This selected image section represents the template that is subsequently matched in the US images captured during the scan. At each (*x*, *y*)-position of the image *I* the similarity with the template *T* is determined by calculating the normalized correlation coefficient:

8 $$\begin{aligned} C_{\textrm{all}}(x,y) = \frac{\sum _{x',y'} (T(x',y') \cdot I(x+x',y+y'))}{\sqrt{\sum _{x',y'}T(x',y')^2 \cdot \sum _{x',y'} I(x+x',y+y')^2}} \end{aligned}$$

Thus, the resulting matrix $C_\mathrm{{all}}(x,y)$ contains a similarity value with the template for each (*x*, *y*) position in the source image. Possible vessel positions are represented by the best matches, which is why the values of $C_\mathrm{{all}}(x,y)$ are sorted in decreasing order and then referred to as $C_v$. The second step in Fig. 2 shows two of these matches as examples, where $C_{v,i}$ represents the *i*th value of the sorted items. Thus, $C_{v,1}$ is considered to be the best match. It is important to prevent incorrect matches in other image regions, as otherwise the robot may be guided away from the target position. Therefore, potential vessel positions are then checked for plausibility with regard to the position $P^{t-1}$ from the previous time step (third step in Fig. 2) using the tracking algorithm in Section “Vessel tracking”.

#### Vessel doppler signal

**Fig. 3:**
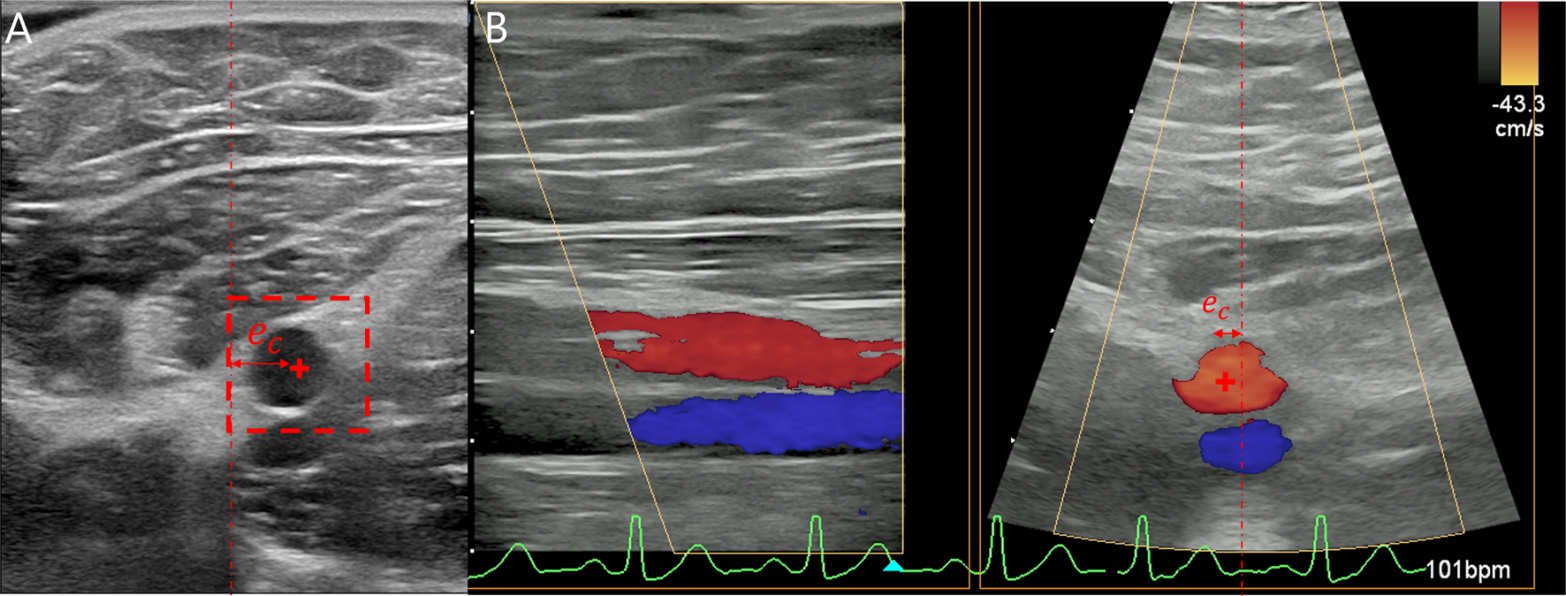
**A** Vessel position determined by template matching. **B** Determination of the vessel position from the Doppler X-Plane US image

The Doppler signal, which is used to visualize blood flow, is of major importance in clinical practice for the diagnosis of vascular diseases, including peripheral arterial disease. Nevertheless, it’s rarely found in robotic US interventions. Since we are using a 3D probe, we can benefit from the X-Plane view of the ultrasound station, which enables the simultaneous visualization of the longitudinal and cross-section of the vessel (see Fig. 3B).

**Fig. 4:**
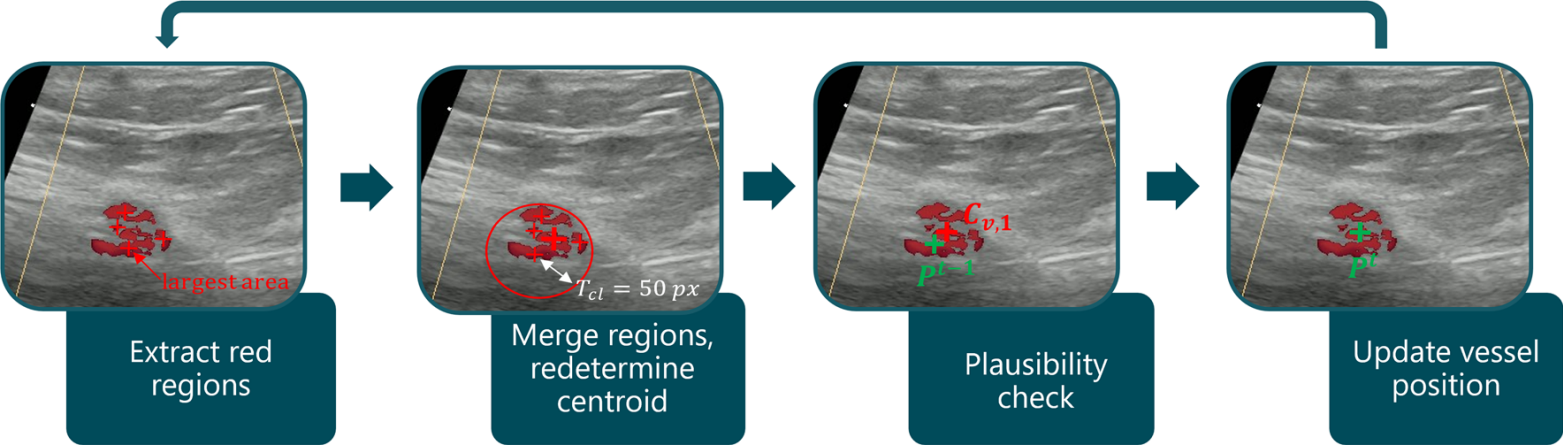
Procedure of doppler vessel tracking. Red regions are extracted and merged into bigger clusters. The centroid of the merged cluster is indicated by the thicker red cross representing the potential vessel position $C_{v,1}$. The vessel’s position is updated following a plausibility check concerning the vessel’s position $P^{t-1}$ of the previous time step

The procedure to find potential artery positions based on the Doppler images is illustrated in Fig. 4.

The Doppler images showing the cross-section of the artery are first converted into the HSV color space and detected flow areas are determined by filtering all red-colored areas (first step in Fig. 4). The values of the corresponding HSV ranges are:

9 $$\begin{aligned}{} & {} H_\textrm{red} \in [0,10] \cup [170, 180] \end{aligned}$$

10 $$\begin{aligned}{} & {} S_\textrm{red} \in [50,255] \end{aligned}$$

11 $$\begin{aligned}{} & {} V_\textrm{red} \in [20,255] \end{aligned}$$

The resulting red areas are sorted according to their size in descending order and referred to as $C_\textrm{all}$. In the next step, they are grouped into clusters if they are close to each other (see Algorithm 2 in the Appendix A). For this purpose, the Euclidean distances between the largest area’s center and the centroids of the remaining areas are calculated. If the distances are smaller than a threshold $T_\textrm{cl} = 50$ px, which in our settings corresponds approximately to the radius of the artery, they are combined, and the centroid is redetermined (see Fig. 4, second step). This merging of red areas is done because the increasing or decreasing blood flow caused by the heart contraction can result in many smaller, unconnected colored areas in the Doppler US image (see Fig. 4). The resulting merged clusters represent potential vessel positions $C_v$ . Here, $C_{v,1}$ represents the largest red cluster and is therefore regarded as the most likely match for the vessel’s position. Finally, $C_{v,1}$ is checked for plausibility in relation to the vessel’s position $P^{t-1}$ from the previous time step.

#### Vessel tracking

The artery to be tracked can now be determined from the potential vessel positions $C_v$ and is described in Algorithm 1, which is based on [17]. Due to the continuous nature of the tracked vessel, the corresponding vessel centers should be close to each other in successive images. Thus, the tracked vessel is updated based on the minimum Euclidian distance between the potential vessel positions $C_v$ in the current image and the detected vessel center $P^{t-1}$ in the last frame. In addition, a maximum distance value $T_d=50$ pixels is defined to avoid incorrect assignments of the vessel center. If the distances of the clustered areas are all above the limit value or no vessel candidate is detected at all, the vessel center of the previous image is retained.

**Algorithm 1:**
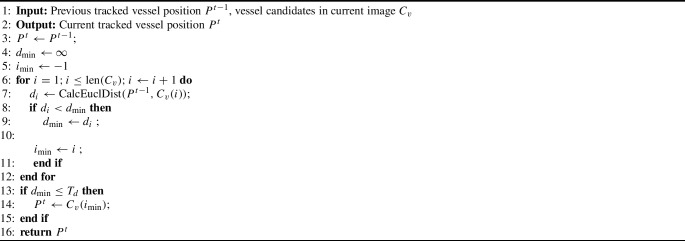
Vessel tracker

### Volunteer study

To validate our method, we performed a volunteer study with seven male and healthy volunteers, in which the femoral artery was scanned by a robot. All volunteers signed informed consent, and the study was approved by the local Ethics Committee of the University of Lübeck (ID2023-216_1). We used a KUKA LBR iiwa 14 R820 redundant robot with seven DoF, a Philips Epiq 7 US station, and the XL14-3 probe. For US data acquisition, the PLUS framework [25] was used in combination with a customized Dicom Network Library provided by Philips, allowing real-time access to image and volume data. As the US station does not have a direct interface to the Doppler signal, we additionally used a frame grabber (Elgato Cam Link 4k) to receive the colored Doppler images from the screen of the US station. The recording frequency was 10 Hz for the Doppler X-Plane images and 25 Hz for the 2D US data used with the template matching approach. The image depth was set to 50 mm , the resulting image spacing was 0.098 $\frac{mm}{px}$. During the study, a physician was present constantly.

The procedure of our volunteer study was as follows: First, while the robot was in gravity compensation mode with the US probe attached to the end effector, the starting point of the scan was determined by the physician. This was the division of the common femoral artery into the profunda femoral artery and the superficial femoral artery. Then two automatic US scans were performed, with each experiment being repeated three times. The maximum scan length of the superficial femoral artery was set at 20 cm. While scanning, either template matching (see Section “Vessel template matching”) or Doppler signal tracking (see Section “Vessel doppler signal”) was used to track the vessel’s center. To position the probe appropriately, the distance $e_c$ between the center of the US image showing the cross-section plane and the tracked vessel center was calculated (see Fig. 3). If this distance $e_c>50$ px, the probe was moved by the robot in the direction of the vessel. Otherwise, the probe was moved forward along the vessel.

During the probe’s movement, a force of 5 N was applied in the direction of the probe’s *z*-axis to keep the probe in contact with the skin. The probe’s orientation was held constant. The control parameters were determined experimentally, with damping factors $d_i$ representing the diagonal entries of the damping matrix *D* in Eq. 7 set to 5 *Nms*. Moreover, the robot’s joint impedance controller was parameterized with a stiffness of 500 $\frac{Nm}{\textrm{rad}}$ and a damping ratio of 0.9.

## Results and discussion

For evaluation purposes, the probe contact forces as well as the vessel tracking accuracy were investigated. The actual vessel centers of the recorded US data were labeled by another physician.

### Vessel tracking accuracy

**Fig. 5:**
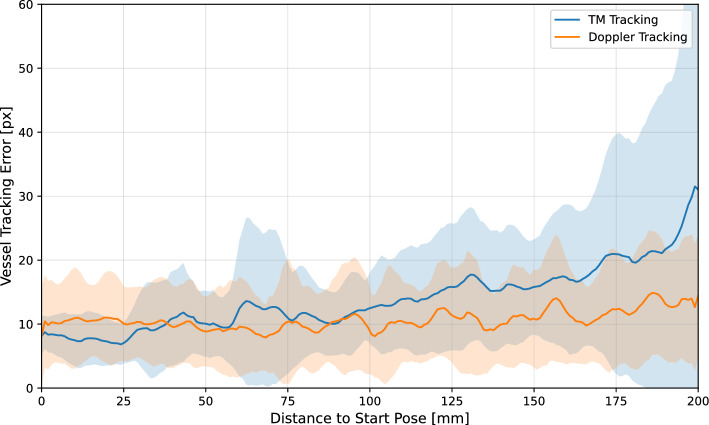
Accuracy of vessel tracking over the distance to the start pose of all scans performed. The blue line represents the mean tracking error using the template matching approach, the orange line represents the mean error using the Doppler approach. The corresponding standard deviations are indicated by the colored areas

The total mean vessel tracking error of all scans performed is 13.9 (± 6.4) px or 1.36 (± 0.63) mm for the template matching approach and 10.7 (± 3.1) px or 1.05 (± 0.3) mm for the Doppler tracking approach. This results in a 23 % lower error when using Doppler tracking. The difference is even more apparent when the tracking accuracy is evaluated in relation to the distance to the start pose of the scan. Figure 5 shows the error between the determined vessel center and the labeled ground truth , depending on the distance to the scan’s start pose.

The blue line represents the average error and the area highlighted in light blue the standard deviation of the template matching approach. The orange line represents the respective results for the Doppler tracking approach. It can be seen that the tracking errors at the beginning of the scan are at about the same level of approx. 10 px. After a scan length of approx. 100 mm, the error of the template matching approach increases, while the error of the Doppler tracking remains constant. The more distance covered, the less accurate the template matching becomes, which can also be recognized by the very large standard deviation towards the end of the scan. The reason for this is that the vessel geometry changes over the distance traveled. This makes the template matching, which uses the vessel cross-section at the start of the scan as a reference image, less reliable. Especially towards the end of the scan, it is difficult even for a physician to recognize the vessel cross-section. This is where analyzing the blood flow using the Doppler signal is a great advantage, as the vessel can still be detected. It should therefore also be noted that due to the poorer visibility of the vessel towards the end of the scan, less accurate labeling could influence the results, especially of the template matching approach. Another advantage of vascular tracking using Doppler signals is the ability to differentiate between veins and arteries based on the visualized blood flow color. This means that false detection of purely image-based methods (e.g. template matching , or neural networks trained on US images) due to the similarity between artery and vein can be avoided. For future work, a combination of both methods could be considered, which combines the advantages. In some cases, no Doppler signal can be detected because the probe is not optimally aligned, even though the vessel is visible. Furthermore, in patients with severe peripheral arterial occlusive disease, the femoral arteries may no longer be sufficiently perfused, making it difficult or even impossible to obtain a Doppler signal. The combination of template matching and Doppler signal could then help to follow the course of the vessel into the periphery. To include the change in the vessel cross-section over the scan, the template should be repeatedly updated.

Some related work described in the literature achieved slightly higher vessel tracking accuracies of 0.89 mm in [13] and 0.38 mm in [18]. However, their validation was done on phantoms, and the neural networks used for recognition were trained with data from the same phantom. In addition, the scan distance in our experiment is 20 cm instead of 14 cm in [13] and [18], and thus significantly larger. Especially in the last part of the scans, the leg vessel is more difficult to see, which reduces the accuracy of vessel recognition in the realistic subject data.

### Contact force accuracy

**Fig. 6:**
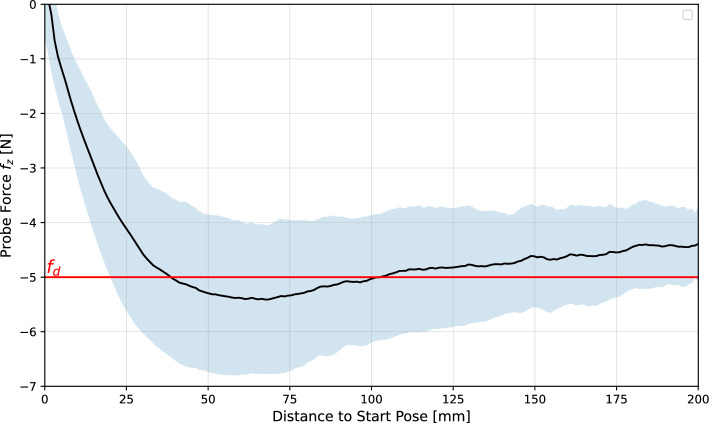
Contact forces $f_z$ acting in the direction of the $^pz$-axis of the probe over the distance to the start pose for all scans. The mean forces are shown as a black line, and the corresponding standard deviations are highlighted in light blue. The value of the desired contact force $f_d$ is shown as a red line

The forces acting on the end effector in the direction of the $^pz$-axis of the probe for all the scans performed are shown in Fig. 6.

**Fig. 7:**
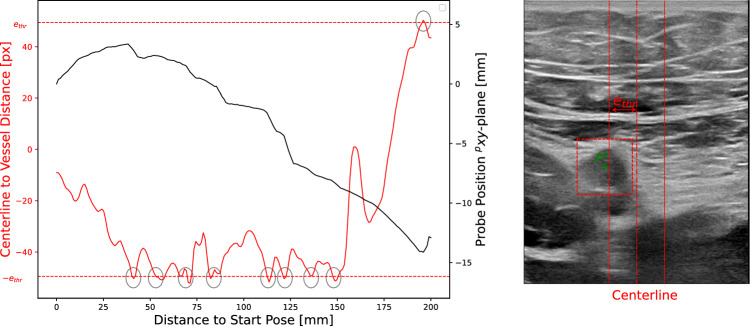
The diagram on the left shows the movement of the US probe in the transversal direction to the vessel’s cross-section in black for an experiment. The distance $e_c$ between the centerline of the US image and the detected vessel center is represented in red. Exceeding the threshold $e_{\textrm{thr}}=50$ px is indicated by the grey circles. The right-hand side of the figure shows an example of an US image in which the centerline (dot-dashed red line) to vessel ($P^t$) distance was higher than the defined threshold $e_{\textrm{thr}}=50$. This results in a corrective movement of the probe to keep the vessel centered

The forces were measured using the torque sensors in the robot’s joints and the force estimation model provided by the manufacturer. It can be seen that after the start of the scan, the contact force increases until the desired value of 5 N is reached. The average measured force then decreases slightly until it approx. reaches 4.4 (± 0.7) N at the end of the scan, which is 0.6 N below the desired target value. The deviation can be explained by inaccuracies in the robot model. Friction and hysteresis effects can lead to inaccuracies in the estimation of internal joint torques, particularly at slow speeds and low external forces acting on the end effector. In addition, the gravity due to the weight of the probe and the probe’s cable was neglected. The inaccuracy of the estimation of the internal torques then also leads to an inaccurate estimation of the external torques $\tau _{ext}$, which are used for the robot’s force control (see Eq. 7). This could affect the controller shown here, as no Cartesian force measurement is used as feedback, which might be further investigated in future work.

### Vessel centering

To keep the vessel centered during movement, the position of the probe must be adjusted. Figure 7 shows an example of the movement of the US probe in the transversal direction to the vessel’s cross-section in black. Moreover, the distance $e_c$ between the center of the US image and the detected vessel center is shown in red. During this scan, the threshold $e_\textrm{thr}=50$ px is exceeded nine times, which is shown by the grey circles. Each time the threshold is reached, the probe is moved in the opposite direction, which is illustrated by the step-like black curve. This shows that the robot follows the detected vessel cross-section. Therefore, the probe can be guided along the vessel. However, it must be noted that there are many corrective movements. This is because the compensation movement stops as soon as it falls below the threshold value. If the vascular curvature continues, another correction is required shortly afterwards, and so on. To reduce the number of required corrective movements, a further threshold value $e_\textrm{thr,ad} < e_\textrm{thr}$ can be introduced in future work. A commanded compensation movement (triggered by exceeding $e_\textrm{thr}$) then continues until the distance between the image center and the vessel falls below this threshold $e_\textrm{thr,ad}$.

### Scanning and processing times

The mean total scan time was 44.7 s for the template matching approach and 37.2 s for the Doppler tracking approach. The lower scanning times for the Doppler tracking approach are due to more precise tracking, reducing false detections and unnecessary robot movements. The acquisition of standard 2D US images took on average 40 ms (25 Hz), while the acquisition of X-Plane Doppler data took 100 ms (10 Hz), due to technical specifications of the hardware used. The template matching approach for vessel tracking required 12 ms on average, while processing and tracking Doppler images needed 10.5 ms.

From a clinical perspective, there are no fixed guidelines regarding the duration of an ultrasound scan. Rather, the divergence between individual examiners is very strong and could be significantly reduced with the help of robotic ultrasound. An average sonographic examination of the arteries of both legs is stated in patient information forms to take around 30-60 min [26, 27]. Even though a direct comparison with our scan times is limited as patient preparation (e.g., undressing, applying gel) was not included, the measured robotic ultrasound times are well below current clinical routine, highlighting the system’s potential for increased efficiency.

## Conclusion

In this work, we presented a robotic system for automatic scanning of the leg artery (superficial femoral artery). We included Doppler Ultrasound to track the artery, which has proven to be an effective option. Compared to the existing methods in the literature that use neural networks for vessel detection (e.g. [17, 18]), the approach we have shown has two major advantages: Firstly, no training data or labeling is required. In particular, to cover different US device settings and different patient-specific vessel morphologies, an extremely large amount of training data would have to be available to ensure robust vessel detection performance. Recording this data is difficult to achieve, especially if data from patients suffering from vascular diseases is required. Secondly, the outputs of our vascular tracking can be easily understood as there is no black box behavior. This would be particularly useful for future fully automated robotic vascular screening applications, as patient safety must always be ensured. Looking to the future, it may be also possible to use robot-assisted ultrasound for safe and precise navigation during peripheral endovascular interventions, with reduced radiation exposure. Additionally, Doppler US can provide real-time visualization of hemodynamic parameters, allowing for improved intra-interventional success monitoring compared to traditional angiography.

## Figures and Tables

**Algorithm 2:**
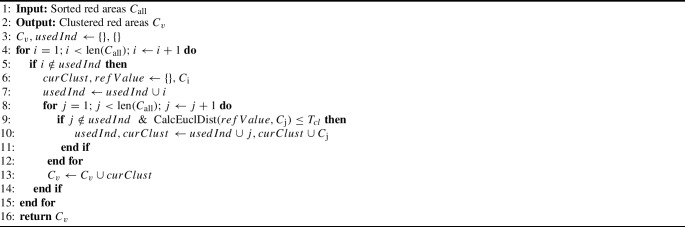
Clustering and merging red regions
